# Variability among *Cucurbitaceae* species (melon, cucumber and watermelon) in a genomic region containing a cluster of NBS-LRR genes

**DOI:** 10.1186/s12864-017-3529-5

**Published:** 2017-02-08

**Authors:** Jordi Morata, Pere Puigdomènech

**Affiliations:** Molecular Genetics Department, Center for Research in Agricultural Genomics, (CSIC-IRTA-UAB-UB), Campus UAB, Edifici CRAG, Bellaterra (Cerdanyola del Vallès), Barcelona, 08193 Spain

## Abstract

**Background:**

*Cucurbitaceae* species contain a significantly lower number of genes coding for proteins with similarity to plant resistance genes belonging to the NBS-LRR family than other plant species of similar genome size. A large proportion of these genes are organized in clusters that appear to be hotspots of variability. The genomes of the *Cucurbitaceae* species measured until now are intermediate in size (between 350 and 450 Mb) and they apparently have not undergone any genome duplications beside those at the origin of eudicots. The cluster containing the largest number of NBS-LRR genes has previously been analyzed in melon and related species and showed a high degree of interspecific and intraspecific variability. It was of interest to study whether similar behavior occurred in other cluster of the same family of genes.

**Results:**

The cluster of NBS-LRR genes located in melon chromosome 9 was analyzed and compared with the syntenic regions in other cucurbit genomes. This is the second cluster in number within this species and it contains nine sequences with a NBS-LRR annotation including two genes, *Fom1* and *Prv*, providing resistance against *Fusarium* and Ppapaya ring-spot virus (PRSV). The variability within the melon species appears to consist essentially of single nucleotide polymorphisms. Clusters of similar genes are present in the syntenic regions of the two species of *Cucurbitaceae* that were sequenced, cucumber and watermelon. Most of the genes in the syntenic clusters can be aligned between species and a hypothesis of generation of the cluster is proposed. The number of genes in the watermelon cluster is similar to that in melon while a higher number of genes (12) is present in cucumber, a species with a smaller genome than melon. After comparing genome resequencing data of 115 cucumber varieties, deletion of a group of genes is observed in a group of varieties of Indian origin.

**Conclusions:**

Clusters of genes coding for NBS-LRR proteins in cucurbits appear to have specific variability in different regions of the genome and between different species. This observation is in favour of considering that the adaptation of plant species to changing environments is based upon the variability that may occur at any location in the genome and that has been produced by specific mechanisms of sequence variation acting on plant genomes. This information could be useful both to understand the evolution of species and for plant breeding.

**Electronic supplementary material:**

The online version of this article (doi:10.1186/s12864-017-3529-5) contains supplementary material, which is available to authorized users.

## Background

The evolution of genes related to pathogen resistance in plants has been the object of intense research for different reasons. These genes are probably related to plant adaptation to different environments where they are confronted with evolving pathogens. These are genes that have also been under selective pressure on domestication, as pathogen resistance is one of the most important traits for plant breeders in many crops. Among these genes, those coding for the NBS-LRR class of proteins have attracted a significant amount of research because they have been associated with effector-triggered immunity, an important component of plant resistance to pathogens, as has recently been reviewed [1]. They are also interesting examples of the evolution of plant gene sequences: the mechanisms that produce genome variability, such as single nucleotide polymorphisms and copy number variation that may occur even without large genome duplication events, appear to act upon these sequences in those species that have been studied.

With the completion of genome sequences of major plant species, it appeared that the structure of gene families with similarity to resistance genes were very variable when comparing different species. In a recent study in angiosperms, the evolution of the lineages of different classes of NBS-LRR genes indicates the importance of whole genome duplication events [2]. The number of genes coding for these proteins varies considerably between species and it has been shown that, in some species, these genes occur in clusters that may be the result of amplification of the gene families. This is the case, for instance, in well studied species such as rice [3].

The case of *Cucurbitaceae* has attracted attention in this respect as they have been shown to contain a reduced number of sequences belonging to the family of NBS-LRR proteins compared to other plants. This has been observed in the main Cucurbit species sequenced so far, such as cucumber [4], melon [5] and watermelon [6], where the numbers (104, 89 and 54, respectively), have been found to be significantly lower when compared to species having a similar genome size, such as those belonging to the Prunus family [7]. In peach, for instance, with a smaller genome than the three cucurbit species studied, more than 400 NBS-LRR sequences have been identified [7]. The report of a correlation between a high number of NBS-LRR genes and the existence of miRNAs controlling their expression [8] is a further indication of the importance of controlling the activity of these genes for the fitness of the species. These observations indicate how variability in the structure and expression of NBS-LRR genes may be involved in the adaptation of plants to different environments. It has also been shown that, at least in melon, a group of sequences coding for these proteins is a hotspot of variability [9]. As an example, a detailed study of a large cluster of NBS-LRR genes in chromosome 5 of melon has shown a high interspecific and intraspecific variability in the number of genes at this location [10]. It was of interest to study whether this observation could be generalized to homologous gene families and to other Cucurbit species. In melon, a second large cluster of NBS-LRR has been observed in chromosome 9, with two major resistance genes (*Fom1* and *Prv*) that have been characterized [11]. The analysis of the variability of this cluster in melon and in the related Cucurbit species was the object of the present study.

## Results and discussion

It has been observed in melon, as in other cases [12–14], that many of the genes coding for NBS-LRR sequences are located in clusters within the genome [5]. These clusters in melon are among those with the highest variability in the genome in terms of presence/absence of genes [9]. As a possible mechanism for adaptation of plants, this has prompted our interest in analysing these clusters in detail in melon and in the syntenic regions of the two other Cucurbit species whose genome sequences are available (cucumber and watermelon).

The largest of the NBS-LRR gene clusters in the melon genome is in chromosome 5 and includes the gene *Vat*, responsible for aphid resistance [15]. The genome of this region in melon has been sequenced and the variability of the genes in this cluster has been compared with other cucurbits [10]. Another cluster of sequences coding for NBS-LRR proteins have been reported in chromosome 9 [5]. There is particular interest in this cluster as it contains two genes, *Fom1* and *Prv*, that have been shown to be responsible for resistance to *Fusarium* and Papaya ring-spot virus, respectively. These genes have been identified from a molecular and genetic point of view and have been found to have an unusual head-to-tail structure [11]. It was therefore of interest to study whether the level of sequence variability in this cluster was similar to that observed in chromosome 5, both between cucurbit species and within these species. In order to answer this question, we carried out a bioinformatic comparative analysis of this region in the different genomic sequences available in melon. This analysis was extended to the syntenic regions of cucumber and watermelon.

**Fig. 1:**
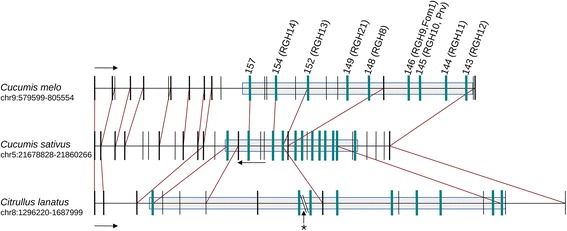
Syntenic relationships between the resistance gene clusters in melon, cucumber and watermelon. Genes are represented by *vertical lines*, in *blue* if they are NBS-LRR genes. NBS-LRR clusters are highlighted in *grey boxes. Red lines* indicate syntenic regions. Direction of transcription of all genes is left to right, except for cucumber which has been reoriented for practical reasons. Chromosome 9 region from melon spans 221 kb from gene MELO3C022168 to MELO3C022141. In cucumber, the syntenic region in chr5 spans 176 kb, from gene Cucsa.237600 to Cucsa.237270 (right to left). Finally, the chromosome 8 region in watermelon (384 kb) includes genes from Cla012442 to Cla012422. The gap of 110 kb in watermelon (asterisk) is composed basically of Ns

Figure 1 shows the structure of the cluster of chromosome 9 and it is compared to the syntenic regions in cucumber and watermelon. Genes with similarity to NBS-LRR proteins (using RGH nomenclature and the numbering in the melon genome) and the other annotated genes are schematically indicated. This sequence corresponds to that reported for part of the cluster previously described [16] but extended with data from the published melon genome sequences [5]. The two sequences correspond to Piel de Sapo melon varieties as whole genome sequencing was with the DHL92 double haploid line of a cross between a Piel de Sapo and a Korean variety, and some regions correspond to each of the parental lines. The region analyzed corresponds to a Piel de Sapo fragment [5]. It appears that the cluster has nine TIR-NBS-LRR genes, which is less than the cluster in chromosome 5 previously studied, that contains more than 20 genes, depending on the melon variety. A number of transposable elements have been observed between the genes and within an intron of the *Prv* gene [16]. In Fig. 1, the melon sequence is aligned with the corresponding syntenic loci of cucumber and watermelon.

When comparing the syntenic areas in melon and cucumber, the length of the genome region of cucumber is shorter, a fact that may correlate with the difference in genome size of the two species: 450 Mb in the case of melon and 350 in the case of cucumber. Nevertheless, the number of genes in the cluster, surprisingly, is much greater in cucumber according to the annotation in the reference genomes published. In the syntenic region located in chromosome 5 of this species, at least 12 of the sequences annotated as NBS-LRR in the reference cucumber genome [4] can be observed. In the case of watermelon, the number of sequences homologous to the melon genes is similar to that in melon but lower than in cucumber. The density of genes in this watermelon region appears to be lower than in melon. However that may be an effect of the quality of the available watermelon sequence, as, for instance, a large insertion between two NBS-LRR genes could be an artefact of the sequencing methods employed.

**Fig. 2:**
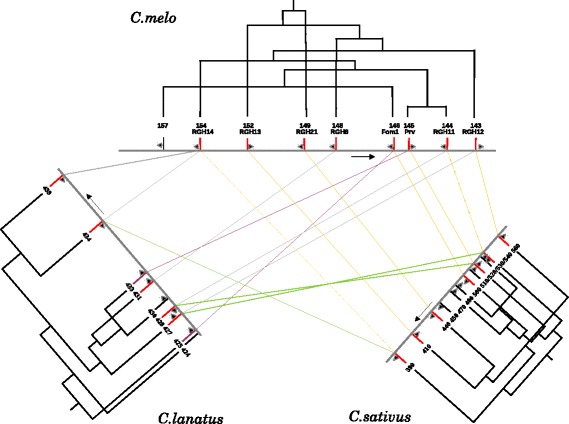
Cluster of resistance genes in melon, phylogenetic relationships and orthology with cucumber and watermelon. Resistance genes are represented by *vertical lines*, in *red* if they have an ortholog in any of the other species, or *black*. Only resistance genes are shown for clarity. Direction of genome assemblies are left to right, except for cucumber which has been reoriented for practical reasons. The transciption direction that is opposite is marked with a *black arrow* next to the gene. Phylogeny was with NBS domains (see text). Branch lengths would give information on the phylogenetic relationships between genes but here the original trees were modified for graphical reasons. *Purple bars and lines* between watermelon and melon are orthologous relationships found in Plaza 3.0

The NBS-LRR sequences of the cluster in chromosome 9 from melon were compared with the corresponding sequences in cucumber and watermelon and a phylogenetic analysis of the sequences between the three species and within each species was carried out. The tree shown in Fig. 2 indicates a possible pathway of how the sequences could be generated in the three species as a result of this analysis. The alignment was compared with that published on the PhylomeDB [17] and Plaza websites [18]. The alignments essentially agree: the exception is in the Plaza system of genes homologous to *Fom1* and *Prv* in watermelon, shown in a different colour in the figure. However the two genes in melon and cucumber are both in the opposite orientation, indicating that they were generated before divergence of the two species. In the other cases, complex patterns of relations, including genes being expressed in opposite directions, were observed. It should be noted that a complete transposon of the CUMULE family [19] was present near *Fom1*, and this may indicate an independent origin of this sequence under the influence of the transposon. Amplification of the gene clusters clearly occurs independently in the different species, in particular in cucumber, as will be discussed later.

**Fig. 3:**
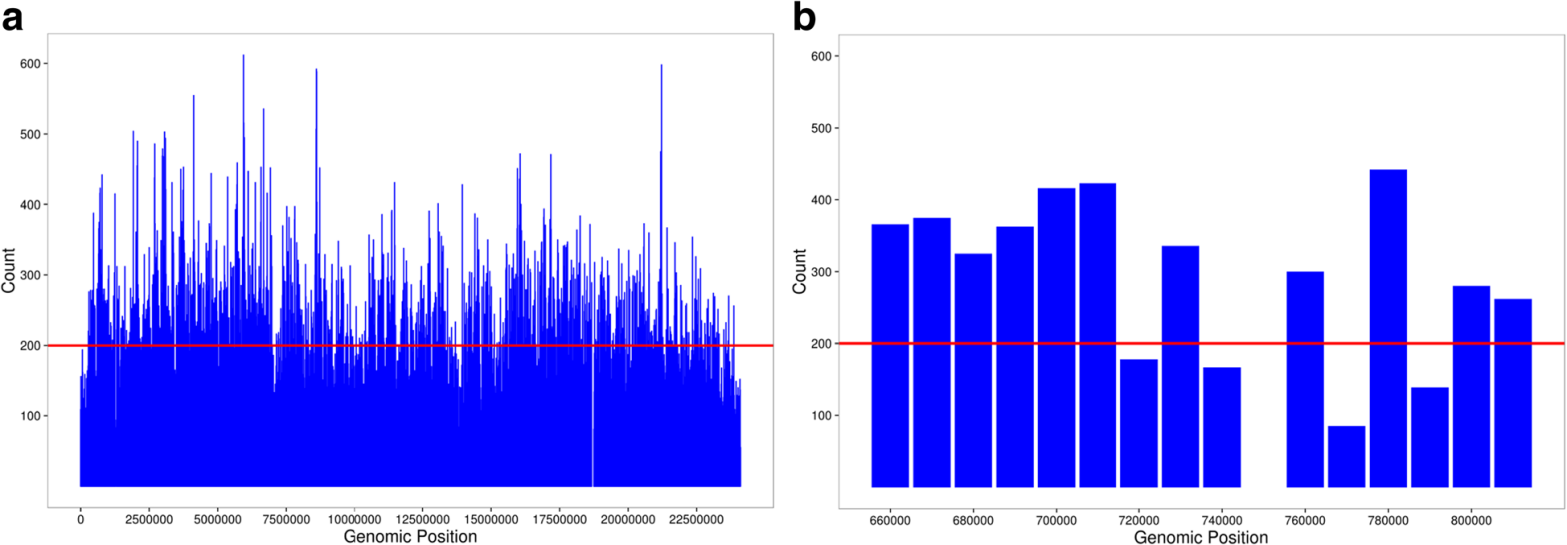
SNP distribution in melon resistance gene cluster. SNP count along chromosome 9 (**a**) and chromosome 9 region (**b**) in melon against six varieties [23]. The *red line* is//indicates the mean number of SNPs per 10 kb bin. The gap in (**b**) is due to Ns

A number of genome sequences are currently available for melon varieties, and they have been used for analysis of intraspecific variability within the species. They have been particularly useful for studying variations in the number of genes present in clusters [9]. In a similar analysis, the reference genome sequence was compared. Single nucleotide polymorphisms, and copy number were recorded along chromosome 9 (Fig. 3). These polymorphisms were unevenly distributed along the chromosome, with an average value around 200 polymorphisms per 10 kb. The value of the genome interval containing the NBS-LRR recorded was very similar. No change in the structure of the cluster was observed in the melon varieties analysed, in contrast to the significant number of presence/absence of genes observed in the cluster of NBS-LRR genes in chromosome 5 [9]. A specific difference was observed between melon varieties in the gene MGH9 that corresponded to Fom1. The cluster of NBS-LRR sequences in chromosome 9 of melon contained two gene sequences that have been shown to be responsible for two genes providing resistance to pathogens, *Fom1* and *Prv* and it has been reported that they have an unusual structure [11]. When analysing the sequence of these two genes (shown in Fig. 1), in the published genome sequence of the DHL92 double haploid line, a unique insertion was found that corresponds to the cultivated Piel de Sapo variety. The insertion was in one of the introns of the gene. Searching in the REPET annotation [20], it was found to contain a complex pattern of several incomplete transposable elements.

A significant difference was observed in the syntenic locus in chromosome 5 of the genome of cucumber. Between the sequences with similarity to those in melon, a number of new sequences belonging to the same family of genes were observed. In this region, 12 sequences with similarity to NBS-LRR genes were found, while only nine were present in melon. Upon further examination, it appeared that the new sequences also coded for NBS-LRR proteins, but were more similar to each other than to the melon genes. These sequences were interspersed with the previous gene sequences (see Fig. 1). A possible hypothesis on how these sequences were generated is based on phylogenetic analysis (Fig. 2). Specific amplification of the cluster in this locus appears to have occurred in cucumber, which has a smaller genome than melon. The generation of new NBS-LRR sequences could have occurred in the different species at specific loci after the separation of the cucurbits during the speciation process. It was of interest to study whether there was variability in the sequences in this cluster in chromosome 5 of cucumber, by comparing the sequences of the two genomes published so far. This result is given in Additional file 1: Figure S1. The two genomic sequences were compared to each other and to the structure of the locus proposed by Lin et al., [21]. The fragmented structure of the genes from the published cucumber genomes made it difficult to accurately compare the two sequences, but no major rearrangement were observed between them.

**Fig. 4:**
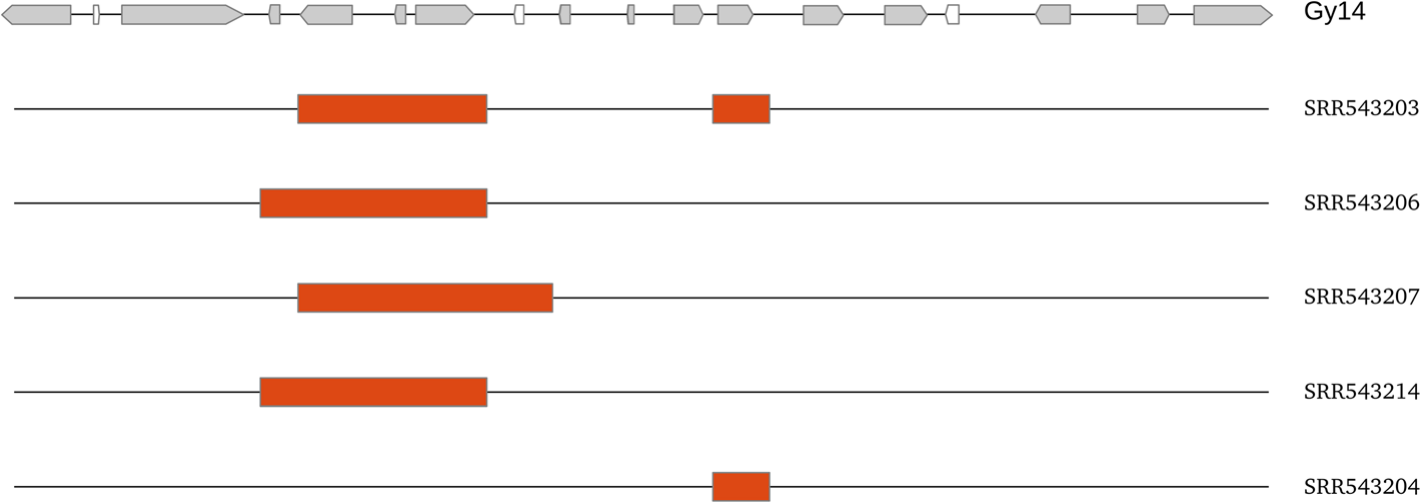
PAV polymorphisms in cucumber chromosome 5 cluster of NBS-LRR genes. *Orange boxes* indicate fragments absent in Indian cucumber varieties compared to the Gy14 reference sequence, where NBS-LRR genes are shown in *grey*

Nevertheless, the study was extended to the other cucumber genomic sequences that have been published [22]. This is a collection of 115 sequences and they have been compared with the reference cucumber genome. It appears that, in a subset of accessions corresponding to Indian harwickii cucumber varieties, a deletion can be observed indicating that presence-absence mechanisms are also active in cucumber, as has been observed in melon. Varieties where PAV polymorphisms are observed are presented in Fig. 4 and the full set of varieties are listed in Additional file 2: File S3. It is interesting to note that four of these PAV polymorphisms include tandem *Fom1* and *Prv* orthologous genes, thus indicating the relevance of genomic variability of these clusters in establishing resistances to pathogens.

## Conclusions

In the *Cucurbitaceae* family, the number of genes coding for the NBS-LRR group of proteins, which are generally related to disease resistance, is consistently lower than in other plant families, and a large proportion of these genes are present in clusters. It has previously been shown that the largest cluster in melon on chromosome 5 is one of the most variable regions in the genome of this species, in terms of presence and absence of genes [9]. Examination of a smaller gene cluster in chromosome 9 showed that the variability in terms of single nucleotide polymorphisms was within the average range observed in the whole chromosome. Apparently there is no major change in the number of genes in the cluster or in the structure of the cluster itself, at least in those melon varieties whose genome has been resequenced to date [23].

In the syntenic region of the two other cucurbit species, cucumber and watermelon, whose genomes have already been sequenced, a cluster of NBS-LRR gene sequences have also been found. The most interesting observation in this cluster was that, while there were no changes in the number of genes in melon, the number of NBS-LRR genes in cucumber was found to be amplified when the locus was compared with the syntenic one. In thesespecies, the cluster was larger than in melon, in spite of the smaller size of the genome. In cucumber, a number of genes conferring resistance to pathogens have been found in chromosome 5, although not in the region of the cluster except a QTL of pathogen resistance to powdery mildew located in this region [24]. Therefore it is probable that there are genes active in providing pathogen resistance in this cluster. Unfortunately the quality of the published sequences of the cucumber genome does not allow the variability in this region to be studied. However when a large number of cucumber genomes is analyzed the absence of a number of genes in the cluster is observed in some varieties from Indian origin. These varieties are included in the hardwickii subgroup of cucumber varieties that clearly separates from other varieties after the analysis of their genomes (see Supplementary Figure 6 in ref. 36). It has to be taken into account that the genetic bases of cultivated cucumber is relatively low and therefore the level of variability is expected to be lower than in other species, including melon.

It may be concluded that clusters of genes coding for proteins having the NBS-LRR features in cucurbits have specific strategies of variability in different regions and between species. It has to be remembered that these are species that have not undergone recent whole genome duplications and that contain a low number of sequences with similarity to the family of resistance genes when compared with other plants. Amplification of NBS-LRR gene numbers have occurred in one locus in chromosome 9 in the case of melon and in another one in the syntenic chromosome 5 in the case of cucumber. This leads to considering that the adaptation of plant species to changing environments may be based upon the variability that may occur at any location in the genome and that has been produced by any of the mechanisms of sequence variation acting on plant genomes.

## Methods

The protein sequences (MELO3C022143 - MELO3C022157), described as a cluster of resistance genes in chromosome 9 of melon [5], were manually inspected and re-annotated with Augustus [25] and FGENESH [26], with *Arabidopsis* as the model organism. For each of the 9 resistance genes in the cluster, a protein sequence was decided based on the output of the re-annotation, the protein sequences described in BAC sequencing studies of the region [16], and taking into account the analysis and criteria (exon structure, domain composition, sequence integrity, etc.) of a study of resistance genes in several *Cucurbitacea* [21]. No additional resistance genes in the cluster were found after scanning the DRAGO database [27] and running a chromosome-wide prediction of NBS, LRR and TIR domains with hmmscan [28] against Pfam profiles database [29].

For watermelon and cucumber (Gy14 and Chinese Long 9930) protein fasta sequences and annotation gff3 were collected from ICUGI [30] and Phytozome [31]. All were downloaded on January 15th, 2015. Cucumber scaffolds where assembled in linkage groups based on a previously published, high resolution genetic map [32].

The phylogeny of the resistance genes for each species was analysed with the NBS domain sequence (Pfam ID: PF00931) predicted with hmmscan and the “One Click” option of the phylogeny.fr web server, based on MUSCLE alignments, PhyML phylogenic analysis and TreeDyn tree rendering [33].

The synthenic regions between each pair of species were determined with MCScanX [34], as described elsewhere [10]. The orthologous relationships between each pair of species was calculated with InParanoid4.1 [35] with default parameters, bootstraping and no outgroup analysis. Further parsing and filtering was done with in-house scripts. Blastp was used for double-check prediction for watermelon orthologous pairs. Melon-cucumber and melon-watermelon orthologous pairs were verified with the PhylomeDB 4 [17] and Plaza 3.0 database [18], respectively.

For annotation of transposon elements in melon, the REPET package v2.2 [20] was used. The SNPs and indels for seven resequences of melon with respect the reference genome [5] have been previously published [23]. The insertion at the first intron in MELO3C022145 gene was confirmed to be unique with respect to previously published sequences [11, 16].

In order to check for presence/absence variation in cucumber, we retrieved 115 published resequences [36] of several varieties, publicly available at ENA [22] (Study: PRJNA171718). The fastq files were trimmed and filtered with skewer [37] and aligned to the Gy14 reference genome with bwa mem [38]. Large deletions were detected with Delly [39]. PAVs were retained when ten or more paired end reads with a minimum mapping quality of one supported the deletion.

## Additional files

Additional file 1: Figure S1. Distribution of the resistance gene cluster in chromosome 5 in the published genomes of cucumber: 9930 (upper) and Gy14 (middle). Dashed lines represent identical regions. Differences are due basically to Ns stretches. Partial and pseudogene annotation (bottom) according to [21]. (PDF 70 kb)
Additional file 2: File S3. Detailed list of PAV polymorphisms in cucumber chromosome 5 cluster of NBS-LRR genes. Orange boxes indicate PAV polymorphisms. (XLS 45 kb)
Additional file 3: File S1. List of the resistance genes in melon, cucumber and watermelon used in this study, along with the gene code, resistance gene class and position in the genome. Protein fasta sequences are available in Additional File 4. Melon gene nomenclature has three codes: gene code [5], resistance gene code [11, 16] and gene name [11]. These genes are shown in Fig. 2. LG = Linkage Group. (XLS 11 kb)
Additional file 4: File S2. Protein fasta sequences of the resistance genes in melon, cucumber and watermelon used in this study. Melon fasta sequences have a resistance gene code [11, 16] and gene name [11]. (TXT 23 kb)
