# Supplementary figures and images for: Variability among Cucurbitaceae species (melon, cucumber and watermelon) in a genomic region containing a cluster of NBS-LRR genes
Source: BMC Genomics. 2017 Feb 8;18:138. doi: 10.1186/s12864-017-3529-5 (PMC5299730; doi:10.1186/s12864-017-3529-5)

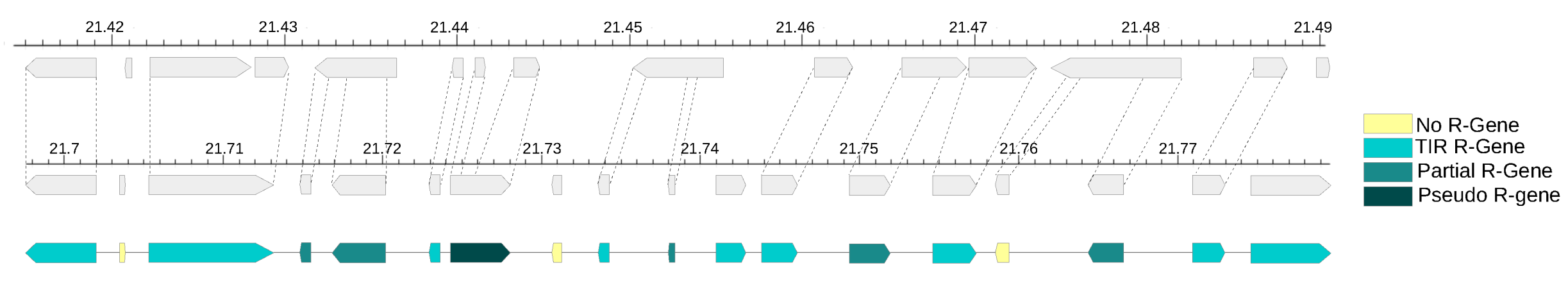

Supplement: Additional file 1: Figure S1. — Distribution of the resistance gene cluster in chromosome 5 in the published genomes of cucumber: 9930 (upper) and Gy14 (middle). Dashed lines represent identical regions. Differences are due basically to Ns stretches. Partial and pseudogene annotation (bottom) according to [21]. (PDF 70 kb) [file 12864_2017_3529_MOESM1_ESM.pdf]
